# Genome-wide mutation spectra of canonical and atypical UV photoproducts in *S. cerevisiae*

**DOI:** 10.1093/nar/gkag482

**Published:** 2026-05-20

**Authors:** Marian F Laughery, Jamison C Haueter, Hailey N Patchett, Piotr A Mieczkowski, Steven A Roberts, John J Wyrick

**Affiliations:** School of Molecular Biosciences, Washington State University, Pullman, WA 99164,United States; School of Molecular Biosciences, Washington State University, Pullman, WA 99164,United States; School of Molecular Biosciences, Washington State University, Pullman, WA 99164,United States; Department of Genetics, Lineberger Comprehensive Cancer Center, University of North Carolina, Chapel Hill, NC 27599, United States; Department of Microbiology and Molecular Genetics, University of Vermont Cancer Center, University of Vermont, Burlington, VT 05405, United States; School of Molecular Biosciences, Washington State University, Pullman, WA 99164,United States

## Abstract

UV exposure causes not only cytosine-to-thymine (C > T) substitutions in dipyrimidine sequences but also many non-canonical mutation classes (e.g. A > T, T > C, and AC > TT). While C > T substitutions are thought to arise from mutagenic bypass of cyclobutane pyrimidine dimers (CPDs), the photoproduct(s) that cause non-canonical mutation classes, which are responsible for key driver mutations in melanoma, are unclear. Here, we use lesion-specific photolyases and whole genome sequencing of yeast irradiated with predominately UVB light to dissect the origins of different classes of UV mutations. These data reveal that CPDs are responsible for ∼60% of all UV mutations and cause not only C > T mutations but also a subset of non-canonical mutation classes, particularly in CT sequence contexts. Our data also indicate that UV-induced pyrimidine (6–4) pyrimidone photoproducts (6–4PPs) are responsible for slightly less than half of all UV-induced mutations in yeast and are the primary cause of T > C substitutions in TT sequences and C > A substitutions. Finally, ∼5% of UV mutations are resistant to both CPD and 6–4PP photolyases, and these are comprised of A > T and AC > TT substitutions in purine-containing dinucleotides. Collectively, these findings define the photoproducts responsible for different UV mutation classes across a eukaryotic genome and indicate that many A > T and AC > TT substitutions arise from atypical UV photoproducts.

## Graphical Abstract

**Graphical Abstract:**
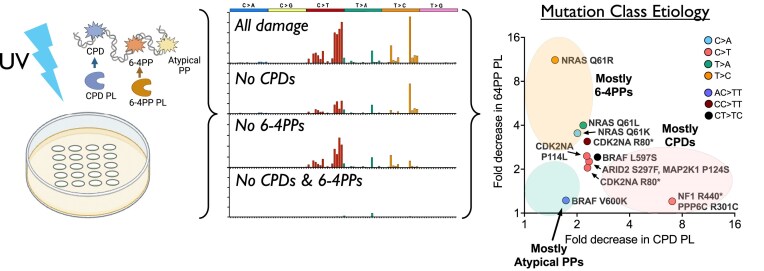

## Introduction

Exposure to ultraviolet (UV) light is the primary cause of skin cancers, such as melanoma, because UV light induces mutagenic lesions in DNA [1–3]. The most common UV-induced lesions are *cis–syn* cyclobutane pyrimidine dimers (CPDs, ∼75%–90% of DNA damage induced by UVB (i.e. 280–320 nm) light) and pyrimidine (6–4) pyrimidone photoproducts (6–4PPs, ∼10%–25% of UVB damage), both of which exclusively form between adjacent pyrimidine bases [4–7]. The formation of each lesion is highly dependent on sequence context [5, 8–10], with CPDs favoring TT, TC, and CT dipyrimidines (i.e. TT > TC > CT > CC), while 6–4PP occur most frequently at TC sequences and least frequently at CT dipyrimidines (i.e. TC > TT > CC >> CT). Helix-distorting photoproducts play a critical role in promoting carcinogenesis, as individuals with defects in nucleotide excision repair (NER), which repairs these photoproducts [5], have a >1000-fold increase in the risk of skin cancer [11].

In mammalian cells and human skin cancers, mutagenic bypass of UV photoproducts causes a preponderance of cytosine-to-thymine (C > T) substitutions in dipyrimidine sequences, which comprise as much as 70%–90% of somatic mutations in sequenced skin cancer genomes [12–14]. However, many of the key driver mutations in melanoma do not fit this mutation signature [15–18]. Instead, the most common driver mutations in melanoma are caused by T > A (i.e. *BRAF* V600E and *NRAS* Q61L), T > C (i.e. *NRAS* Q61R), C > A (*NRAS* Q61K), and AC > TT (*BRAF* V600K) substitutions. Moreover, a number of these driver mutations (e.g. *BRAF* V600E and V600K) occur in a non-dipyrimidine sequence context. We and others have shown that UV exposure in yeast causes not only UV signature mutations (i.e. C > T and CC > TT), but also many of these same non-canonical mutation classes (e.g. T > C, T > A, C > A, and AC > TT), and that these occur in yeast at a frequency significantly higher than in mammalian cells [18–21]. However, the DNA lesion(s) responsible for these non-canonical UV mutation classes is unknown. UV exposure can also induce rare purine-containing photoproducts, such as the thymine-adenine photoproduct (TA-PP) [22–24]. However, to what extent these and other atypical photoproducts contribute to UV mutagenesis remains unclear.

The use of transgenic CPD and 6–4PP photolyases to remove lesions following UV exposure has been instrumental in understanding the contributions of these lesions to mutagenesis [25, 26]. Studies using transgenic photolyases and mutation reporter genes in mouse cells or live mice revealed that CPDs are responsible for the vast majority of UV-induced mutations, while 6–4PPs had a negligible contribution to the UV mutagenesis [25, 26]. However, similar studies in NER-deficient human cell lines yielded conflicting results, with one study finding that CPDs were responsible for nearly all UV-induced mutations [27], consistent with studies in mouse cells, while a second study indicated that 6–4PPs caused nearly half of all UV-induced mutations [28]. This latter study also observed residual T > A substitutions when a UV-irradiated plasmid reporter was repaired using both CPD and 6–4PP photolyases prior to being transfected into human skin cells defective in NER. However, the UV-induced lesion responsible for these T > A substitutions was unclear. Furthermore, previous studies seeking to dissect the individual mutagenic contributions of CPDs and 6–4PPs have largely relied on reporter assays that yield relatively limited numbers of mutations [25–28].

Here, we use WGS to clarify the origins of these mutations by coupling UV exposure with photoreactivation of damage by CPD and/or 6–4PP photolyases, in order to specifically analyze the mutations induced by each UV photoproduct. Analysis of >63 000 UV mutations indicated that CPDs and 6–4PPs both significantly contribute to the UV mutagenesis in yeast, and that these photoproducts induce partially overlapping mutation spectra. Furthermore, we find that noncanonical mutation classes, including T > A substitutions and AC > TT tandem mutations, are enriched in photoreactivated yeast harboring both photolyases, indicating that these mutations are likely derived from atypical UV photoproducts. Our findings clarify the mutagenic potential of CPDs, 6–4PPs, and atypical photoproducts in yeast, and shed light on potential origins of similar mutation classes found in melanoma driver genes.

## Materials and methods

### Strain construction

All yeast strains used in this investigation carry *rad16∆* background deletions; their genotypes and the plasmids and oligonucleotides used in constructing them can be found in Supplementary Table S1. Haploid and diploid strains containing endogenous yeast CPD photolyase (CPD PL) but deficient in global genomic-NER (GG-NER; *rad16∆*) are MP072 and YML155, respectively, and have been previously described [20]. The “No PL” (i.e. no photolyase) *rad16∆phr1∆* haploid strain YML496 was made by CRISPR–Cas9 mediated deletion of *PHR1* using a previously described single-plasmid CRISPR–Cas9 system that we previously developed [29, 30]. Here, pML159 was constructed by ligating the Cas9-containing pML104 plasmid [29, 30] with hybridized OML327 and OML328 oligonucleotides, and the resultant plasmid was transformed into MP072 along with the single-stranded homologous template oligonucleotide OML340. Mutant isolates were selected on synthetic complete media lacking uracil (SC-Ura) and successful candidates were verified with internal and external primers via PCR analysis.

No PL passaging was performed in two diploid strains, YML481 and YML482. YML481 was constructed by CRISPR–Cas9-mediated deletion of *PHR1* in YML155 using the Cas9-containing plasmid pML159 and the single-stranded homologous template oligonucleotide OML340. Mutants were confirmed through phenotypic analysis (i.e. loss of viability on plates after UV exposure and UVA photoreactivation relative to control cells) and PCR screens for loss of the gene. *PHR1* is situated on the subtelomeric region of chromosome XV, and upon analysis of whole genome sequenced isolates, it was discovered that YML481 was lacking ∼35 kb approximately downstream of position 1 057 000 on the chromosome. Inspection of known genes and putative ORFs in this region revealed that it contains neither essential genes nor those known to be involved in DNA repair; however, an alternate No PL strain (i.e. YML482, see below) was constructed to verify the passaging results.

YML482 was constructed in YML478, a *rad16∆* diploid yeast strain rendered *leu2^−/−^* by CRISPR–Cas9-targeted disruption of the *LEU2* gene in YML155; this strain was generated by transformation with pML160 and OML343. Mutants were confirmed by phenotypic screening for inability to grow on synthetic complete medium lacking leucine (i.e. SC-Leu). The deletion of *phr1∆* was achieved by CRISPR–Cas9-mediated deletion of *PHR1* using pML159 and OML340. PCR and whole genome sequencing (WGS) analysis confirmed that the deletion was restricted to the *PHR1* locus in this strain. *rad16∆* yeast strains carrying *Drosophila melanogaster* dPhr(6–4) and *PHR1* (i.e. “Both PL” yeast) were constructed by transformation of the integrating vector pML157 containing dPhr(6–4) into haploid (i.e. MP072) or diploid (i.e. YML478) yeast and selection on SC-Leu media. The pML157 plasmid was constructed by isolation of the *Drosophila* photolyase gene from p64PLA5 (Addgene #67284) via NheI/XhoI digestion and gel purification, and ligation with the p405TEF1 plasmid (Addgene #15968), which had previously been SpeI/XhoI digested and gel purified. Successful integration was confirmed by screening with primers OML336 and OML330 which were specific to the *TEF1* promoter region and inside the dPhr(6–4) ORF, respectively. The resulting strains were YML497 (haploid) and YML487 (diploid).

Haploid *rad16∆* yeast expressing only *Drosophila* 6–4PP photolyase (i.e. *rad16∆ phr1∆* + *dPhr(6–4)*) were generated by CRISPR–Cas9-mediated deletion of *PHR1* in YML497 to produce YML498. The *phr1*∆ mutant was confirmed through PCR analysis. For diploid yeast, pML157 was transformed into YML482 and integration of the plasmid was selected through growth on SC-Leu plates. Presence of *dPhr(6–4)* was confirmed through amplification with the OML336/OML330 primers.

### Canavanine resistance assays

To test the frequency of UV-induced mutations induced in irradiated and photoreactivated yeast, overnight cultures of haploid strains grown in YPDA medium were diluted in water, poured into petri dishes (Falcon #351007), and exposed to 150 J/m^2^ of predominately UVB light with a UVP CL-1000M midrange crosslinker (Analytik Jena), which contains bulbs with peak UV emission at 302 nm. The spectrum of the UVP CL-1000M UVB source provided by the manufacturer indicated negligible amounts of UVC light; however, our subsequent investigation using a Spectrilights ILT940-UV mini-spectrophotometer indicated that the UV emission spectrum is predominantly UVB, but does contain a small but significant proportion of UVC light. Following exposure, lids were placed on the Petri dishes and they were incubated for 40 min under UVA lamps (Spectroline EA-160), which have a peak emission at 365 nm. Following both exposures, cells were pelleted by centrifugation, resuspended in YPDA medium, and incubated at 30°C with shaking overnight or at least 12 h. Cells were then diluted in water and plated on SC-Arg + 0.006% (w/v) canavanine plates to measure the frequency of canavanine-resistant (Can^R^) *can1* mutant cells, or SC plates to assess total number of viable cells. Control experiments were also conducted in which cells were exposed to forty minutes of UVA but no UVB radiation. Plates were incubated at 30°C for 3–4 days before counting. A minimum of six replicates was performed for each yeast strain, and the frequency of canavanine resistant colonies was calculated as number of colonies growing on SC-Arg + canavanine plates divided by the number of colonies on SC plates, adjusted for the dilution factor of the latter, as described [20].

### UV passaging assays

Overnight cultures of diploid yeast strains were diluted in water and spotted on YPD or YPDA plates, allowed to dry, and exposed to 150 J/m^2^ of predominately UVB light (see above) in a UVP CL-1000M midrange crosslinker (Analytik Jena) with the lids off; exposure to 150 J/m^2^ required ∼4 s. The lids were then replaced, and plates were placed under UVA lamps (Spectroline EA-160) for approximately 40 min (yielding a total dose of roughly ∼50 kJ/m^2^ of UVA light), before incubating them at 30°C. Plates were incubated until growth was apparent, typically overnight or up to two days. Using sterile toothpicks, cells were picked from each spot, resuspended in water, and re-spotted onto fresh YPD or YPDA plates. UVB/UVA exposures were then repeated and passaging continued in this way until a total of 15 UVB/UVA exposures had been achieved. Fifteen exposures of UV light were chosen based on our previous studies indicating that this yielded a sufficient number of UV-induced mutations in yeast, and to match the experimental design and overall UV dose utilized in these studies [19–21]. After cell growth from the final incubation was apparent, spots were struck for isolation to YPD/YPDA plates, and single colonies were selected from each. Genomic DNA was extracted from isolates using bead beating and phenol:chloroform:isoamyl alcohol extraction and submitted for WGS, as previously described [19, 20].

### Bioinformatics analysis

UV-induced mutations were identified from WGS of passaged isolates were called using CLC Workbench, version 10.0, as previously described [19, 20]. Briefly, paired fastq files resulting from WGS of passaged isolates were imported as Illumina sequences into CLC Genomics Workbench and mapped to the sacCer3 reference genome using the Map Reads to Reference tool available in the NGS Core Tools module. Following this, the Basic Variant Detection tool in the Resequencing Analysis module was employed on the mapped files to compile a table of variants in the isolates relative to the reference genome. Here, the ploidy value was specified as 2 (i.e. diploid). Additionally, parameters were set to require a minimum threshold of 10 reads and a minimum of 45% allelic fraction for calling variants. After variant calling was completed, Microsoft Excel was used to filter out variants occurring in the mitochondrial chromosome as well as those occurring >2 times within the entire passaging replicate (i.e. >2 identical mutations occurring in any two isolates for all strains passaged within the replicate experiment). The latter was performed to screen out mutations that may have arisen in the yeast strain prior to the passaging experiment. The total number of sequenced isolates for each strain that passed quality control checks and were used for subsequent analysis are indicated in Supplementary Table S2.

Analyses of the change in mutation frequency between yeast expressing photolyases and those without it were performed using Welch’s unpaired *t*-test. To analyze the enrichment of mutations occurring on the non-transcribed strand (NTS) versus the transcribed strand (TS) of genes, trinucleotides were normalized by their occurrence across the yeast genome, and significance was determined using a chi-squared test with the Bonferroni correction. The coordinates for transcribed genes were obtained from [31], and transcriptional strand asymmetry of UV-induced mutations was analyzed as previously described [19, 20].

### Alkaline gel quantification of UV-induced lesions

To determine the total number of CPDs, 6–4PPs, and atypical photoproducts produced by the cumulative dose given to the passaged yeast isolates, YML482 (i.e. the “No PL” diploid yeast strain) was grown overnight in YPD culture medium. The next day, cells were subcultured into fresh medium and grown to a final OD_600_ of 0.6–1.1 before harvesting by centrifugation and resuspending in sterile water. Cells were then poured into a tray and irradiated with an AnalytikJena CL-1000M, which produces predominately UVB radiation, set to a dose of 2250 (i.e. 150J/m^2^ × 15) according to the manufacturer’s specifications. A portion of the cells were also reserved and left unirradiated to constitute “No UV” controls. Cells were then pelleted and their genomic DNA was immediately isolated by bead beating and phenol:chloroform: isoamyl alcohol extraction as previously described [19, 20]. Purified DNA pellets were resuspended in either 10 mM Tris pH 7.5–8.0 or 1× TE buffer (10 mM Tris pH 7.5–8, 0.1 mM EDTA) and incubated with 1 μl RnaseA/100 μl DNA for 30–50 min at 37°C. To quantify the number of CPD lesions, isolated genomic DNA was digested with T4 endonuclease V (T4 endo V, NEB) in the manufacturer’s provided buffer at a concentration of ∼1 μl enzyme per 25 μg of purified DNA quantified Nanodrop spectrophotometry for approximately 1.5 h at 37°C. To quantify the number of 6–4PPs and atypical photoproducts, isolated genomic DNA was first incubated with purified CPD photolyase (CPD PL, Biotechne) at a concentration of 1 μl enzyme per 33–38 μg DNA in ∼1× photolyase buffer (10 mM NaCl, 5 mM Tris pH7.5, 2 mM DTT, 0.1 mM EDTA, and 5% glycerol) under UVA lamps (Spectroline EA-160) for 2 h, followed by phenol:chloroform:isoamyl alcohol extraction and ethanol precipitation of the DNA. Recovered DNA was then digested with purified UVDE (0.68 mg/ml, purified by S.A.R., essentially as previously described [20]) at a concentration of 1 μl/25 μg of input DNA (as determined by Nanodrop Spectrophotometry) for 45 min at 55°C in reaction buffer at a final concentration of ∼36 mM HEPES pH 6.5, 180 mM NaCl, and 1.8 mM MnCl_2_.

DNA samples that had been digested with T4 endo V, CPD PL/UVDE, or no enzyme (as a control) were loaded onto 1.2% alkaline gels and run under alkaline conditions (50 mM NaOH and 1 mM EDTA) at 30 V for 18–19 h. Gels were neutralized in a neutralization buffer consisting of 1 M Tris, pH 7.5, 1.5 M NaCl, stained with SYBR Gold (Invitrogen) in 0.5× Tris Borate EDTA (TBE) buffer, and destained in Millipore water before imaging on a Typhoon FLA biomolecular imager (GE Healthcare). Images were analyzed with ImageQuant TL, and lane profiles were exported into Microsoft Excel, wherein the median intensity of each lane was used to determine the average fragment size based on running distance relative to bands of a HindIII digested λ phage ladder (NEB). An approximate number of CPDs (from T4 Endo V digested samples) and 6–4PPs + atypical photoproducts (from CPD PL treated and UVDE digested samples) was calculated assuming a Poisson distribution of the fragments, and the No Enzyme control samples were subtracted from these to account for background cutting, essentially as previously described [32]. Results shown are derived from four independent UV exposures.

## Results

**Figure 1.**
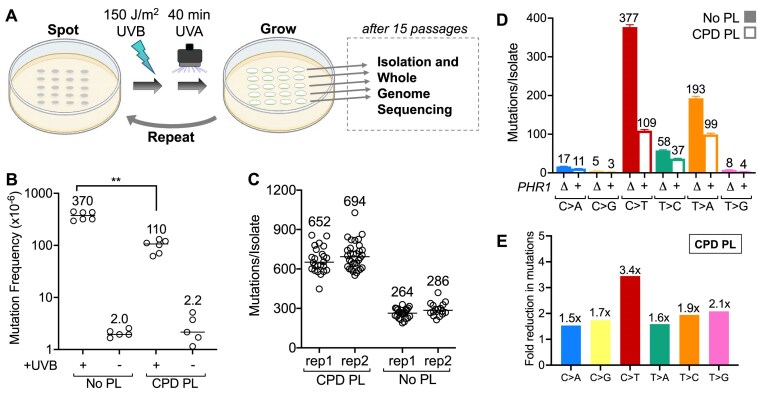
UV-induced mutation classes caused by CPDs. (**A**) Schematic of yeast passaging protocol, referred to as UVB/UVA passaging. Yeast were spotted onto rich medium plates, exposed to predominately UVB light, followed by photoreactivation for 40 min with UVA light, and then allowed to recover under normal culture conditions. This process was repeated 15 times. After 15 serial passages, genomic DNA was extracted from a single clonal isolate from each spot and submitted for WGS. Figure is reproduced and adapted under terms of the Creative Commons Attribution 4.0 International License. [18] 2024, The Authors, published by *Advanced Genetics*. Elements of the image were created in BioRender. Laughery, M. (2026) https://BioRender.com/na1z2u7. (**B**) *CAN1* mutation frequency of *rad16∆* yeast irradiated with 150 J/m^2^ UVB (or unirradiated No UVB control) followed by photoreactivation with 40 min of UVA. Yeast strains either carried the gene for endogenous CPD photolyase (“CPD PL”) or harbored a *phr1∆* deletion (“No PL”). *CAN1 m*utation frequencies were calculated as the number of colonies growing on canavanine plates divided by the number growing on control plates, with both adjusted by their respective dilution factors. Median values are listed above each experimental data set. **P = 0.0022 based on Mann–Whitney test. (**C**) Total mutations per isolate identified by WGS for two independent UVB/UVA passaging experiments with *rad16∆ phr1∆* (i.e. No Photolyase [No PL]) and *rad16∆ PHR1* (i.e. CPD Photolyase [CPD PL]) yeast). Median mutation counts are indicated for each replicate. (**D**) Analysis of the SNV mutation classes identified by WGS resulting from each strain (i.e. *rad16∆ phr1∆*, No Photolyase [No PL] and *rad16∆ PHR1*, CPD Photolyase [CPD PL]) yeast). Results are reported as average mutations per substitution class, error bars represent standard error of the mean (SEM) values. (**E**) Fold reduction of mutation classes observed in CPD PL yeast versus No PL yeast. Values were obtained by dividing the average number of No PL mutations per isolate for each class by the average number in the CPD PL isolates.

To characterize mutations induced by UV-induced CPD lesions, we exposed diploid yeast (*Saccharomyces cerevisiae*) cells with wild-type *PHR1*, which encodes the endogenous CPD photolyase gene (“CPD PL” yeast), or *phr1∆* knockouts (“No PL” yeast) through 15 independent doses of 150 J/m^2^ of predominately UVB light (see Materials and methods), followed by 40 min of photoreaction with UVA light (Fig. 1A). This timeframe of UVA photoreactivation (i.e. 40 min) was chosen based on preliminary UV sensitivity experiments, and our previous study measuring CPD repair by photolyase in yeast [33], to ensure that essentially all CPDs would be repaired in the CPD PL yeast cells. The yeast cells were also GG-NER deficient (i.e. *rad16*∆), since rapid removal of one class of UV damage (i.e. CPDs) could indirectly promote more efficient NER of other classes of UV photoproducts (i.e. 6–4PPs and atypical photoproducts) in GG-NER competent cells. The *rad16*∆ cells showed moderate sensitivity to the 150 J/m^2^ UVB dose, consistent with our previous report [19], but photoreactivation for 40 min with UVA light rescued this sensitivity in yeast cells containing the endogenous CPD photolyase, but not in the *rad16*∆ *phr1*∆ (No PL) control (Supplementary Fig. S1). While the *rad16*∆ cells showed sensitivity to predominantly UVB light, they were not sensitive when exposed to 40min of UVA light alone (total dose of ∼roughly 50 kJ/m^2^; see Supplementary Fig. S1). After each exposure, cells were allowed to recover in rich media so that any unrepaired damage (i.e. 6–4PPs, atypical photoproducts, etc.) could be converted into mutations via DNA replication, and the resulting UV-induced mutations were identified by the WGS.

Pilot experiments using haploid *rad16*∆*phr1*∆ (No PL) yeast cells indicated that exposure to 150 J/m^2^ of UVB light resulted in a significant increase in the frequency of canavanine resistant (Can^R^) colonies due to mutations in the *CAN1* gene (Fig. 1B). This mutation frequency was ∼190-fold higher than control yeast cells not exposed to UVB light (i.e. 40 min of UVA alone; Fig. 1B). Photoreactivation in cells with CPD PL (i.e. *rad16*∆ *PHR1*) rescued the UV sensitivity of the *rad16*∆ cells (Supplementary Fig. S1) and resulted in ∼3.5-fold fewer UVB-induced Can^R^ mutants relative to the No PL cells (Fig. 1B). However, there were still ∼50-fold more Can^R^ mutants than cells not exposed to UVB light (Fig. 1B), indicating that the vast majority of mutations are caused by UVB exposure, even when CPDs are subsequently repaired by photolyase.

WGS was used to characterize mutations in individual clonal isolates of diploid *rad16*∆ yeast cells with (CPD PL) or without (No PL) endogenous CPD photolyase after 15 exposures of 150 J/m^2^ of predominately UVB light followed in each case by 40min of UVA photoreaction (Fig. 1A). Analysis of the resulting WGS data yielded a median of 652 mutations per isolate in the No PL strain and 264 mutations per isolate in the CPD PL strain, in which CPD lesions were removed by photolyase (see replicate 1 [rep1] in Fig. 1C). A second independent replicate of the UV exposure protocol yielded very similar mutation counts for each strain (i.e. 694 and 285 mutations per isolate for the No PL and CPD PL strains, respectively; see Fig. 1C, rep2), indicating that the results from the UV exposure and photoreactivation protocol were very reproducible. Moreover, the UVB/UVA passaging of No PL yeast resulted in a mutation burden similar to that produced exclusively by exposure of *rad16∆* yeast to the UVB source alone (i.e. 676 mutations/isolate [19]). Analysis of WGS data from the first passaging experiment (rep1) revealed that the original No PL diploid strain harbored a ∼33kb deletion of the end of Chromosome XV that included *PHR1*, suggesting that it was likely incurred during the CRISPR–Cas9-mediated deletion of *PHR1*. Upon inspection of the deleted region, we found few genes of known function, none of which are known to function in damage response other than *PHR1*. The second passaging replicate (rep2) was performed in a reconstructed version of the *rad16∆ phr1∆* yeast strain that did not carry this additional deletion. Statistical analysis revealed that there was no significant difference in the number of accumulated mutations between the two replicates (Mann–Whitney *U* test, *P *= 0.30), and mutations from both replicates were consequently aggregated in subsequent analysis, yielding a total of ∼38 000 mutations in No PL yeast and ∼10 000 mutations in CPD PL yeast. Consistent with the pattern observed in the *CAN1* mutation assay, UVB/UVA passaged CPD PL yeast accumulated less than half the number of mutations observed No PL yeast (Fig 1C), having 40%–41% of the mutation count per isolate in each experimental replicate.

### UV-induced CPDs primarily causes C > T substitutions, as well as other classes of mutations in specific sequence contexts

To clarify how the removal of CPDs influences the spectrum of UV mutations in yeast, we analyzed the number of mutations per isolate in each class of single base substitutions (i.e. C > A, C > G, C > T, T > A, T > C, and T > G) in the No PL and CPD PL data. Analysis of the No PL mutation spectrum indicated that exposure to predominantly UVB light induced not only C > T substitutions, but also abundant T > C and T > A substitutions and occasional C > A substitutions (Fig. 1D), consistent with our previous report [19]. The CPD PL yeast had significantly fewer mutations per isolate than the No PL yeast in each substitution class (*P *< 0.0001 for each class using Welch’s *t*-test; Fig. 1D). As expected, the greatest reduction occurred in C > T mutations (Fig. 1D and E), consistent with previous reports that CPDs are largely responsible for UV-induced C > T substitutions [2, 25]. Nonetheless, CPD PL yeast still had ∼109 C > T mutations per isolate, despite removal of CPDs by photoreactivation, indicating that other UV-induced lesions may also cause C > T substitutions. Moreover, CPD photoreaction also significantly reduced T > C, T > A, and C > A substitutions, albeit to a lesser extent than C > T substitutions (Fig. 1D and E).

**Figure 2.**
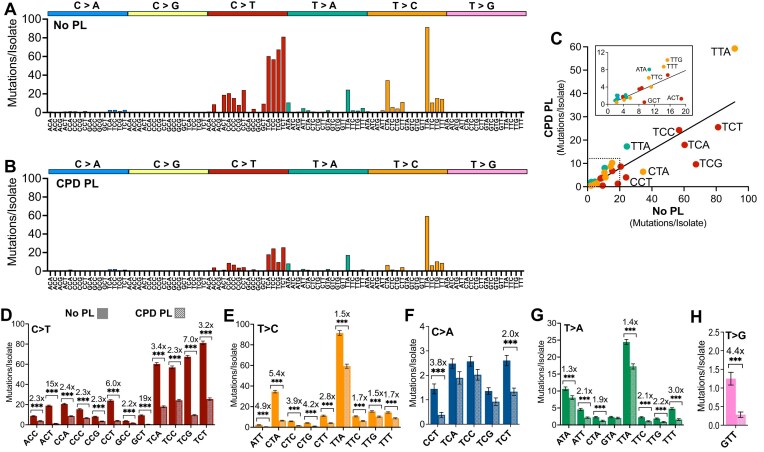
Spectrum of mutations induced by CPDs. (**A**) Mutation spectrum of UVB/UVA passaged *rad16∆ phr1Δ* (i.e. No Photolyase [No PL]) yeast. Mutations per isolate for each mutation class (e.g. C > A, C > G, C > T) are plotted in their trinucleotide context, with the mutation occurring in the middle base. (**B**) Mutation profile of UVB/UVA passaged *rad16∆ PHR1* (i.e. CPD Photolyase [CPD PL]) yeast. Mutations are depicted as described in (A). (**C**) CPD Photolyase (CPD PL) yeast are depleted in C > T mutations for most contexts relative to No PL yeast. Trinucleotide contexts containing with a minimum of 100 total mutations in the No PL strain were plotted for the frequency of each mutation class in CPD PL and No PL isolates. C > A mutations are blue, C > T mutations are red, T > A mutations are green, T > C mutations are orange, and T > G mutations are pink. Inset shows a magnification of the area within the dotted lines. (**D**) Comparison of the average C > T mutations/isolate for trinucleotide contexts in the No PL (solid bars) and CPD PL (shaded bars) isolates. Only trinucleotides with at least 70 cumulative mutations in No PL are shown. Error bars represent SEM values, significance shown was determined using Welch’s unpaired *t*-test. ***P < 0.001. The fold reduction in average mutations/isolate is shown for trinucleotide contexts in which the difference between No PL and CPD PL is statistically significant. The same comparison method was also used to analyze the effect of photoreactivation with endogenous CPD photolyase on (**E**) T > C, (**F**) C > A, (**G**) T > A, and (**H**) T > G mutation classes.

To elucidate how DNA sequence context impacts CPD-induced mutagenesis, we analyzed the number of single base substitutions per isolate in different trinucleotide sequence contexts. In the No PL yeast, UV-induced C > T, C > A, and T > C mutations primarily occurred in trinucleotide contexts containing lesion-forming dipyrimidine sequences, with C > T substitutions being enriched in TCN, CCN, and NCT sequence contexts (with “N” indicating any nucleotide) and T > C substitutions primarily occurring in TTN and CTN sequence contexts (Fig. 2A), consistent with previous results [19, 20]. Comparison with the UV mutation spectrum in CPD PL yeast (Fig. 2B) indicated that the magnitude of the reduction in substitution classes due to CPD removal varied with substitution type. C > T substitutions generally showed relatively strong reductions (e.g. more than most T > C substitutions, Fig. 2C); however, the magnitude of the reduction in C > T and other substitutions significantly varied with trinucleotide context (Fig. 2C–G). For example, C > T mutations were particularly reduced in NCT contexts (e.g. up to 19-fold decrease in the CPD PL), as well as in TCG trinucleotides (7-fold, Fig. 2C and D). In contrast, C > T mutations in CC contexts (i.e. ACC, CCA, CCC, CCG, GCC) showed much smaller reductions in mutation frequency (i.e. <3-fold) in the CPD PL strain (Fig. 2D). A similar trend was seen for T > C substitutions, which showed a much greater reduction in CTN contexts (e.g. up to 5.4-fold decrease) than in TTN contexts (i.e. <2-fold, see Fig. 2E). Less abundant C > A mutations, which primarily occur in TCN sequence contexts, did not show significant decreases in the CPD PL strain except for mutations occurring in YCT contexts (i.e. CCT and TCT, Fig. 2F). UV-induced T > A substitutions showed significant, albeit modest, decreases in CPD PL (Fig. 2G), while T > G substitutions in a GTT context were decreased ∼4.4-fold (Fig. 2H), indicating this latter mutation class is primarily caused by CPDs. Taken together, these findings suggest that CPDs are the major contributor to UV-induced C > T substitutions, but also cause other mutation classes (e.g. C > A, T > A, T > C, and T > G), particularly in CT sequence contexts.

We previously demonstrated that UV-induced mutations in non-photoreactivated *rad16∆* yeast exhibit strong transcriptional strand asymmetry [19, 20], with UV mutations enriched on the NTS of genes and suppressed on the TS. This transcriptional asymmetry is a hallmark of mutations caused by UV photoproducts that block ongoing RNA polymerase II transcription, and therefore trigger efficient transcription coupled-nucleotide excision repair (TC-NER) [20, 34–36]. We wondered whether the UV-induced mutations in the CPD PL yeast isolates also show transcriptional asymmetry, consistent with these mutations originating from mutagenic bypass of other (non-CPD) photoproducts that are repaired by TC-NER.

**Figure 3.**
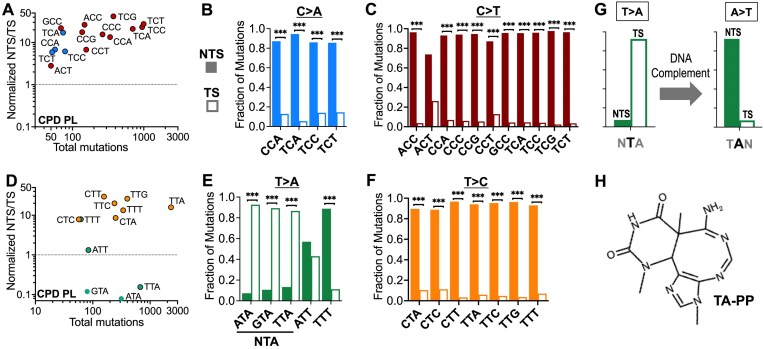
Analysis of transcriptional strand asymmetry of UV mutations in *rad16*∆ *PHR1* CPD photolyase (CPD PL) isolates. (**A**) Plot of the normalized ratio of C > A (blue) or C > T (red) mutations (in different trinucleotide sequence contexts) in the NTS of yeast genes relative to the TS in CPD PL passaged yeast isolates. The normalized NTS/TS ratio is plotted relative to the total mutation count for each trinucleotide mutation class. Only trinucleotide contexts with a minimum of 50 total mutations are depicted. Contexts containing dipyrimidines are indicated with a black outline. (**B** and **C**) Plot of the normalized fraction of (B) C > A (blue) and (C) C > T (red) mutations in different trinucleotide contexts in the NTS (solid bar) and TS (outlined bar) in yeast genes. **P < 0.001 based on Chi-square test with Bonferroni correction. (**D**) Plot of normalized NTS/TS ratio for T > A (green), T > C (orange), and T > G (pink) mutations in different trinucleotide contexts, plotted as described in panel (A). (**E** and **F**) Plot of the normalized fraction of (E) T > A (green) and (F) T > C (orange) in different trinucleotide contexts in the NTS (solid bar) and TS (outlined bar) in yeast genes. **P < 0.001 based on chi-square test with Bonferroni correction. (**G**) Enrichment of T > A mutations in NTA contexts along the TS of genes suggests that causative UV photoproduct is occurring on the complementary strand, resulting in A > T mutations in TAN trinucleotide context. (**H**) Chemical structure of thymine-adenine photoproduct (TA-PP).

To test this hypothesis, we analyzed the transcriptional strand asymmetry of mutations in different trinucleotide contexts in the CPD PL isolates (Fig. 3). This analysis indicated that C > A and C > T mutations were significantly enriched (*P *< 0.001) on the NTS relative to the TS in essentially all trinucleotide contexts (Fig. 3A–C). Likewise, nearly all T > A and T > C mutations showed significant transcription strand asymmetry (Fig. 3D–F). Together, these results suggest that mutations in CPD-depleted yeast (i.e. CPD PL) arise from UV photoproducts repaired by NER. Notably, T > A mutations in NTA contexts exhibited reverse strand asymmetry, with the apparent enrichment of mutations on the TS (Fig. 3D and E). A similar analysis of mutations observed in No PL yeast revealed the same pattern of elevated mutations on the NTS of genes for most mutation classes, but reverse strand asymmetry for T > A mutations in NTA contexts (Supplementary Fig. S2). Due to the specificity of TC-NER for lesions on the TS, our findings suggest that the lesion responsible for T > A mutations in NTA contexts are instead occurring on the opposite strand (i.e. the NTS), resulting in A > T mutations in a TAN context (Fig. 3G). This fits the mutation signature of an atypical TA photoproduct (Fig. 3H), which have previously been biochemically characterized and mapped genome-wide in yeast following UVC irradiation [10, 18, 20, 22, 24, 37, 38]. Taken together, the transcriptional strand asymmetry of UV-induced mutations in CPD PL yeast indicates that they originate from non-CPD photoproducts, including 6–4PPs and atypical TA photoproducts.

### UV-induced 6-4PPs primarily cause T > C and C > A substitutions

Because 6–4PPs are the second most common UV photoproduct, we hypothesized that they are likely responsible for most of the UV-induced mutations in photoreactivated yeast cells with endogenous CPD photolyase (CPD PL). Although yeast lack 6–4PP photolyase, 6–4PP photolyases have been found in a variety of prokaryotic and eukaryotic species, including the fruit fly *Drosophila melanogaster* [39] To test whether 6–4PPs influence the UV-induced mutation spectrum in yeast, we integrated a single copy of the *Drosophila* 6–4PP photolyase gene under the control of the constitutively expressed yeast *TEF1* promoter. The 6–4PP photolyase transgene was integrated in *rad16∆* yeast strains with or without the endogenous CPD photolyase (i.e. *PHR1* WT or *phr1∆*). This yielded yeast strains that only expressed 6–4PP photolyase (i.e. “6–4PP PL” yeast) or that expressed both CPD and 6–4PP photolyases (i.e. “Both PL” yeast).

**Figure 4.**
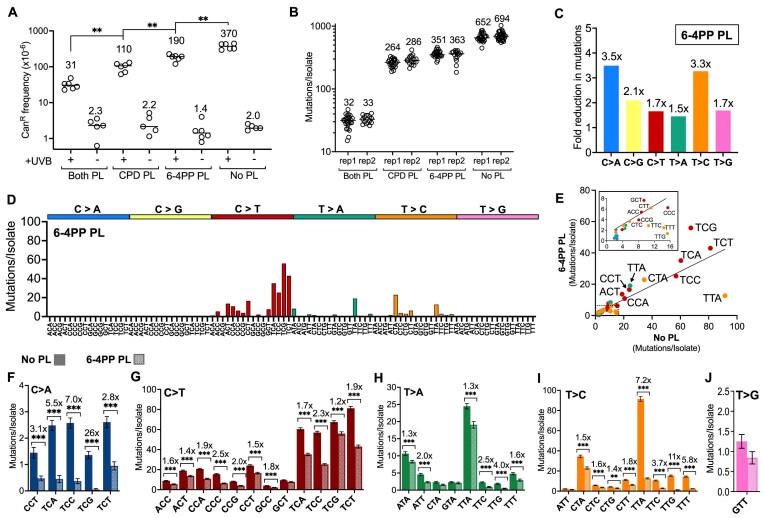
Analysis of UV mutations in yeast caused by 6–4PPs. (**A**) Plot showing frequency of canavanine resistant (Can^R^) *can1* mutants in haploid yeast cells for GG-NER deficient (*rad16*∆) yeast cells with the following genotypes: *rad16∆ phr1∆* (i.e. No Photolyase [No PL]), *rad16∆ PHR1* (i.e. CPD Photolyase [CPD PL]), just the exogenous *Drosophila* 6–4PP photolyase (6–4PP PL, *rad16∆ phr1*∆ *dPhr(6–4)*), or both photolyases (Both PL, *rad16*∆ *PHR1 dPhr(6–4)*). Yeast strains were irradiated with 150 J/m^2^ UVB (or unirradiated No UVB control) followed by photoreactivation with 40 min of UVA. Results show median values of six independent replicate exposures (or at least five for controls). **P < 0.01 based on Mann–Whitney test. Data for CPD PL and No PL are same as Fig. 1B. (**B**) Plot showing the total number of mutations identified by WGS that were accumulated in UVB/UVA passaged No Photolyase (No PL), CPD Photolyase (CPD PL), 6–4PP photolyase (6–4PP PL), and Both Photolyases (Both PL) diploid yeast isolates. Two independent experimental replicates (rep1 and rep2) were performed for each strain. Data for No PL and CPD PL isolates are from Fig. 1C. (**C**) Plot showing the fold-reduction for each substitution class in the 6–4PP PL strain. Values were obtained by dividing the average mutations per isolate values of No PL yeast by the average mutations per isolate values in 6–4PP PL yeast for each substitution class. (**D**) The trinucleotide spectrum of mutations observed in UVB/UVA passaged 6–4PP PL diploid yeast. Bars are colored according to substitution class and depict the average number of mutations per isolate for each trinucleotide context, where the middle base is the one mutated, as indicated above the graph. (**E**) 6–4PP PL yeast are depleted in T > C mutations in TTN contexts relative to No PL yeast. Plot shows mutations per isolate in No PL and 6–4PP PL UVB/UVA passaged strains for trinucleotide contexts in which there were a minimum of 100 mutations in the No PL strain. The color of each dot represents the mutation class in accordance with the mutation scheme shown in panel (C). Inset shows a magnification of the area within the dotted lines. (**F**) Comparison of the average C > A mutations/isolate for trinucleotide contexts between passaged No PL (solid bars) and 6–4PP PL (shaded bars) yeast. Only trinucleotides with at least 70 cumulative mutations in No PL are shown. Error bars represent SEM values, significance shown was determined using Welch’s unpaired *t*-test. ***P < 0.001. The fold reduction in average mutations/isolate is shown for trinucleotide in which the difference between No PL and CPD PL is statistically significant. (**G–J**) Same as panel (F), except for (G), C > T, (H), T > A, (I), T > C, and (J), T > G mutations.

Photoreactivated haploid 6–4PP PL yeast experienced a nearly 2-fold reduction of UV-induced *CAN1* mutants relative to yeast expressing no photolyases (Fig. 4A). Notably, yeast with both photolyases displayed a ∼12-fold reduction of UV-induced mutations, suggesting that CPDs and 6–4PPs together comprise the vast majority of mutagenic UV-induced lesions (Fig. 4A). However, the frequency of Can^R^ colonies in the Both PL strain was >10-fold higher in UV-exposed cells than unexposed (Fig. 4A), indicating that at least some classes of mutagenic UV-induced lesions are not repaired even when both CPD and 6–4PP photolyases are present.

WGS was used to identify mutations in individual clonal isolates of diploid *rad16*∆ yeast cells expressing either the 6–4PP photolyase alone (6–4PP PL) or both CPD and 6–4PP photolyases (Both PL) after 15 exposures of 150 J/m^2^ of predominately UVB light followed in each case by 40 min of UVA photoreaction (Fig. 4B). The 6–4PP PL yeast accumulated slightly more than half of the mutations per isolate relative to the No PL yeast, while yeast cells expressing both photolyases (both PL) accumulated only 5% of the UV-induced mutations relative to No PL yeast (Fig. 4B). These mutation frequencies were highly reproducible, as a second independent passaging experiment resulted in very similar median values for each strain (Fig. 4B). Moreover, adding together the decrease in mutations per isolate in the single photolyase expressing strains (i.e. CPD PL or 6–4PP PL strains) relative to the No PL control roughly matched the overall decrease in mutation count per isolate in the yeast strains expressing both photolyases (Fig. 4B). Collectively, our passaging experiments resulted in ∼13 500 mutation in 6–4PP PL yeast from 38 independent isolates and ∼1200 mutations in Both PL yeast from 39 independent isolates. While the frequency of UV-induced mutations in the Both PL strain was very low (i.e. 32–33 mutations per isolate), this frequency is >10-fold higher than the background rate of mutations in WT yeast in the absence of UV exposure [19], consistent with the results of the *CAN1* mutation reporter (Fig. 4A).

Comparison of the 6–4PP PL mutation spectrum to the No PL control indicated that C > A and T > C mutation classes show the greatest decrease, being 3.5- and 3.2-fold lower, respectively, in the 6–4PP PL isolates (Fig. 4C). C > T substitutions, which showed the greatest fold change in the CPD PL strain (3.4-fold decrease, see Fig. 1E), decreased only 1.6-fold in the 6–4PP PL isolates. Analysis of the trinucleotide spectrum of the accumulated mutations in the 6–4PP PL isolates revealed a striking reduction in T > C mutations in TTN contexts, particularly TTA (Fig. 4D and E). C > A mutations, while infrequent in the No PL yeast spectrum, were reduced to such an extent that they were nearly undetectable in passaged 6–4PP PL yeast. Indeed, C > A mutations were significantly depleted (*P* < 0.001) in 6–4PP PL yeast for all trinucleotide contexts that had at least 70 total mutations in No PL yeast (Fig. 4F). The magnitude of the decrease mutations was greatest in TCN sequence contexts (up to 26-fold for TCG) and least in CTN contexts (Fig. 4F). Most C > T mutation classes were also significantly reduced in the 6–4PP PL isolates, although the magnitude of the decrease was generally <2-fold (Fig. 4G), with the exception of some CC dinucleotide classes (e.g. CCC, CCG, and TCC). A few classes of T > A substitutions were significantly decreased, particularly in the dipyrimidine-containing ATT, TTC, and TTG sequence contexts (Fig. 4H). T > C mutations in TTN contexts were significantly reduced approximately 4- to 11-fold in 6–4PP PL yeast (*P* < 0.001, Fig. 4I); however, the reduction of T > C mutations in CTN contexts was <2-fold, suggesting that 6–4PPs cause few mutations in CTN sequence contexts (Fig. 4I). There was no significant difference in T > G substitutions in GTT contexts (Fig. 4J). In contrast, there were significant depletion of these same mutation classes in CPD PL yeast (Fig. 2), suggesting that CPDs caused most of the residual UV-induced mutations in the 6–4PP PL strain.

Consistent with this hypothesis, nearly all the residual UV-induced mutations classes in the 6–4PP PL strain showed significant transcriptional strand asymmetry (Supplementary Fig. S3). Most mutation classes (e.g. C > T and T > C substitutions in different sequence contexts) showed >10-fold more mutations on the NTS than the TS of these genes. Again, T > A substitutions in NTA sequence contexts (i.e. ATA, CTA, GTA, and TTA) were outliers, occurring much more frequently on the TS than the NTS of yeast genes, as were T > C substitutions in an ATT context (Supplementary Fig. S3). The enrichment of T > A substitutions in NTA sequence contexts on the TS of yeast genes are consistent with our analysis of transcriptional asymmetry in CPD PL strains (Fig. 3) and suggest that these mutations are caused by neither CPDs or 6–4PPs, but rather are A > T substitutions caused by TA photoproducts.

### Atypical UV photoproducts primarily cause A > T substitutions associated with thymine–adenine dinucleotides

**Figure 5.**
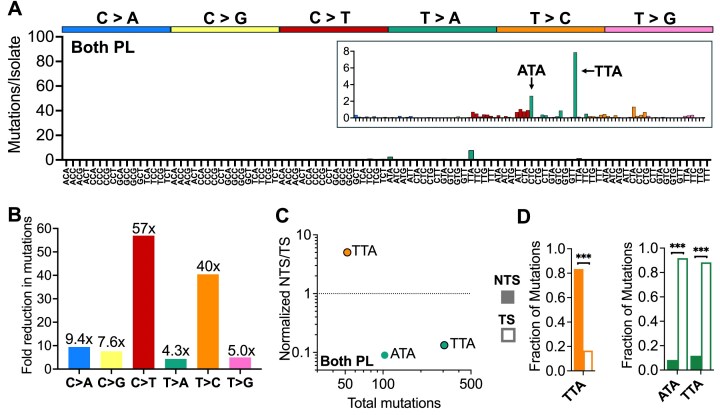
Spectrum of mutations induced by photolyase-resistant atypical UV photoproducts. (**A**) Trinucleotide spectrum of mutations accumulated following UVB/UVA passaging in diploid *rad16*∆ yeast encoding endogenous CPD photolyase and *Drosophila* 6–4PP photolyase (i.e. both photolyase [Both PL] yeast). Bars are colored according to the basic mutations class (e.g. C > A, blue, C > G, yellow, C > T, red, etc.) and depict the average mutations per isolate observed in each trinucleotide context on the same axes previously used in **Figs 2****A, B**, and **4****D** for CPD PL, No PL, and 6–4PP PL yeast, respectively. Inset shows the same mutation spectrum with adjusted *y*-axis values to allow visualization of the contexts of accumulated mutations. (**B**) Plot showing fold reduction in each substitution class, which was calculated as the average mutations per isolate in *rad16∆ phr1∆* (i.e. No Photolyase [No PL]) divided by the average mutations per isolate of both photolyases (Both PL, *rad16*∆ *PHR1 dPhr(6–4)*) yeast. (**C**) Plot showing the normalized ratio of mutations occurring on the NTS relative to the TS of yeast genes for mutation classes with a ≥50 total mutations in a single trinucleotide context in the Both PL isolates. Both PL yeast were severely depleted in total mutations counts and only three contexts fit this criterion: T > A in TTA and ATA (green), and T > C in TTA (orange) contexts. (**D**) Bar graphs of the faction of mutations occurring on the NTS (shaded) versus TS (outlined) for mutation classes shown in panel (C). ***P < 0.001 based on chi-square test with Bonferroni correction.

WGS indicated that the median number of accumulated mutations in individual clonal isolates from a strain expressing both photolyases was 20-fold less than in the No PL yeast control (Fig. 4B). The mutations in the Both PL isolates could presumably reflect mutations arising from residual CPDs or 6–4PPs that escaped photoreactivation repair or alternatively mutations induced by rare atypical UV photoproducts that are not repaired by either photolyase. To address this question we analyzed the trinucleotide mutation spectrum derived from WGS of Both PL isolates. While there were relatively few UV-induced mutations overall, these predominantly were T > A substitutions occurring in ATA and TTA sequence contexts (Fig. 5A). Consistently, T > A substitutions had the smallest decrease in the Both PL strain relative to the No PL control (Fig. 5B and Supplementary Fig. S4), while C > T (∼57-fold reduced) and T > C (∼40-fold reduced) substitutions decreased the most, consistent with the hypothesis that these mutation classes are caused by CPDs and 6–4PPs. The third most abundant trinucleotide mutation class in the Both PL isolates were T > C substitutions in TTA contexts, and together these three were the only trinucleotide mutation classes with at least 50 total mutations in the Both PL yeast isolates.

To test whether these mutations are derived from UV-induced photoproducts, we analyzed their transcriptional strand asymmetry (Fig. 5C). The T > A substitutions were again enriched on the TS, consistent with the hypothesis that these are A > T substitutions arising from in TAN sequence contexts in the NTS of yeast genes (Fig. 5C and D). In contrast, T > C substitutions in TTA sequence contexts were significantly enriched on the NTS of yeast genes (Fig. 5C and D). Since T > C substitutions in TTA sequence contexts are the most frequent class of mutations in non-photoreactivated yeast cells exposed to UV light (Fig. 2), and because these mutations are significantly reduced in the CPD PL and especially the 6–4PP PL strains, we hypothesized that these mutations in the Both PL isolates are caused by residual 6–4PPs (or CPDs) that escaped photoreactivation. In contrast, our data indicate that T > A substitutions in NTA sequence context likely arise from mutagenic bypass of TA photoproducts, a lesion that is presumably resistant to photoreactivation.

### Roles of different UV photoproducts in inducing double base substitutions

**Figure 6.**
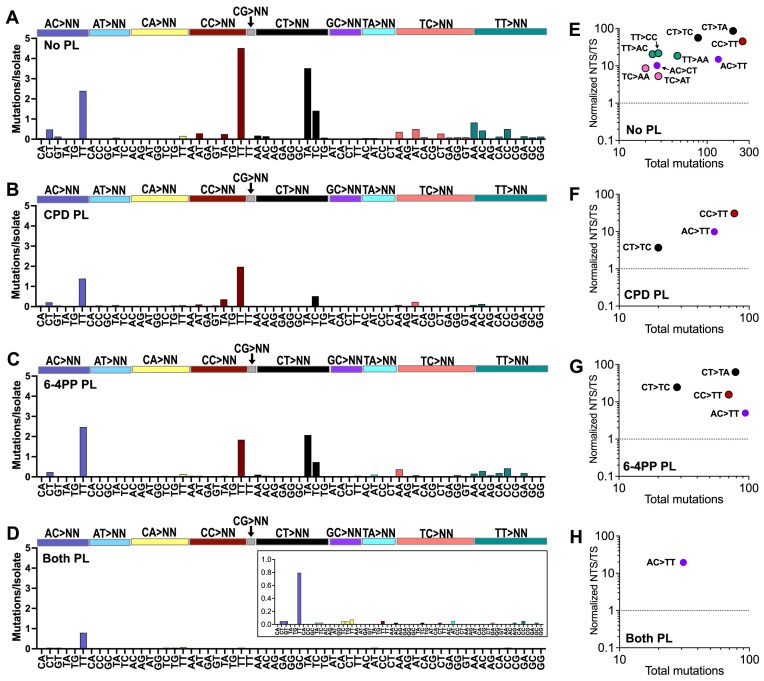
DBSs are enriched in both dipyrimidine sequences and purine-containing AC dinucleotide contexts. (**A**–**D**) DBS spectra of mutations per isolate accumulated in the following GG-NER deficient (i.e. *rad16*∆) strains: (A), No Photolyase (No PL), (B), CPD Photolyase (CPD PL), (C), 6–4PP photolyase (6–4PP PL), and (D), Both Photolyases (Both PL) are shown. Mutations are colored by the dinucleotide context from which they originated (shown above graph), with the *x*-axis labels indicating the resultant sequence to which they were mutated. The substitution classes on the *x*-axis include only mutations classes for which at least one mutation was observed in at least one of the four experimental conditions. (**E**) Scatter plot depicting the normalized NTS/TS ratio for classes in which ≥ 20 total mutations were observed in UVB/UVA passaged No PL yeast. Mutations are colored according to their depiction shown in panel (A), and dipyrimidine contexts are outlined in black. (**F**–**H**) Same as panel (E), except for DBS occurring in (F), CPD PL, (G), 6–4PP PL, and (H), Both PL yeast isolates.

In addition to single base substitutions, UV also induces a substantial number of double base substitutions (DBSs), particularly CC > TT mutations [40, 41]. While less frequent than the single base substitutions analyzed up to this point, DBS were present in substantial numbers in the WGS data derived from the UV-irradiated No PL strain (Fig. 6A), with CC > TT being the most frequent DBS. Only slightly less frequent were CT > TA, AC > TT, and CT > TC substitutions, all of which occurred with a frequency >1 DBS per isolate (Fig. 6A). These latter two classes of DBS (i.e. AC > TT and CT > TC) are of particular interest, since they cause driver mutations in melanoma (i.e. BRAF V600K and L597S, respectively), but the UV photoproducts responsible for these DBS were previously unclear [12, 20].

Analysis of DBS in the CPD PL and 6–4PP PL isolates indicated that DBS in CT dinucleotides (particularly CT > TA mutations) were primarily caused by CPDs, as the overall frequency of these DBS dropped precipitously in the CPD PL strain, but decreased only slightly in the 6–4PP PL isolates (Fig. 6B and C). In contrast, CC > TT mutations averaged ∼2.0 and 1.5 mutations per isolate for CPD PL and 6–4PP PL yeast, respectively, compared to 4.5 CC > TT mutations per isolate in No PL yeast, suggesting that CPDs and 6–4PPs contribute roughly equally to CC > TT mutations. Finally, a variety of TT > NN DBS (e.g. TT > AA, TT > AC, TT > CC, etc.) were primarily reduced in the CPD PL isolates (Fig. 6B and C).

In Both PL isolates, there were few if any CC > TT, CT > TA, and CT > TC substitutions and the frequency of DBS overall was greatly reduced (Fig. 6D), consistent with the hypothesis that these mutation classes are caused by canonical CPD and 6–4PPs. However, AC > TT substitutions still occurred at a considerable frequency and accounted for over half of all DBS in the Both PL isolates (i.e. 31 of 56 total mutations). Analysis of transcriptional strand asymmetry indicated that these different classes of DBS (e.g. CC > TT, CT > TA, CT > TC, AC > TT, etc.) were generally elevated on the NTS of yeast genes in the different yeast strains (Fig. 6E–G), similar to the UV-induced single base substitutions. Notably, the AC > TT substitutions were enriched on the NTS of yeast genes in the No PL control and in each of the photolyase-expressing strains, including the Both PL isolates (Fig. 6E–G and Supplementary Fig. S5), suggesting that these mutations are potentially caused by a helix-distorting UV photoproduct occurring at adenine–cytosine dinucleotides.

### Only a small fraction of UV photoproducts cause mutations

**Figure 7.**
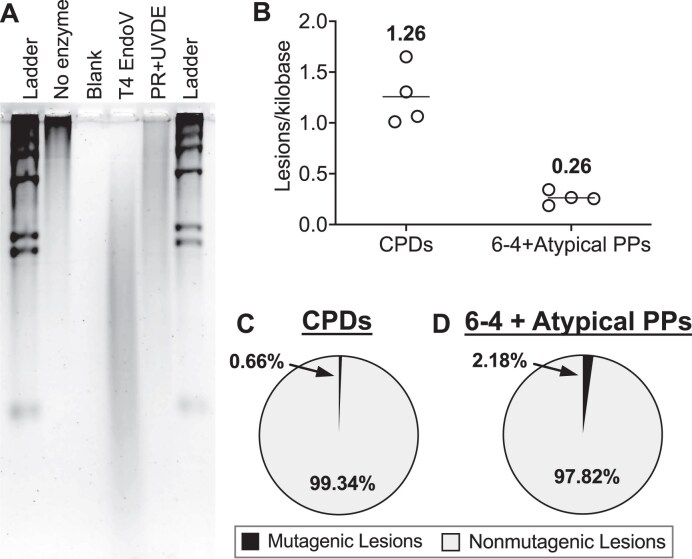
Quantification of UV-induced lesions induced by 2250 J/m^2^ of UVB light, the cumulative UV dose used for passaging experiments. (**A**) Representative image of alkaline gel electrophoresis of UV-irradiated genomic yeast DNA. DNA was either run without enzyme digestion (“No enzyme”), or after digestion with T4 endonuclease V (“T4 EndoV”) to quantify CPD lesions, or after incubation with CPD photolyase followed by UV damage endonuclease (UVDE) digestion (“PR + UVDE”) to quantify 6–4PPs and atypical photoproducts. The signal in each lane was used to determine the average fragment size relative to a HindIII digested **λ** phage ladder (“Ladder”), and this was used to calculate the average number of lesions per kilobase of purified genomic DNA. (**B**) Quantification of the results obtained from electrophoresis of four independent UV exposures; the values listed above are averages of each data set. (**C**) Graphical depiction of the calculated percentage of CPDs that become mutations in *rad16∆ phr1Δ* (i.e. No Photolyase [No PL]) yeast. Values were calculated by dividing the number of mutations attributed to CPDs (deduced from subtracting the median mutation count of CPD PL passaged yeast from the median count of No PL yeast) by the average number of CPDs formed in the cell. The latter was determined by multiplying the lesions/kilobase quantified in panel (B) by the genome size and the number of DNA strands (i.e. 1.26 lesions/kilobase × 24 140 kbp/diploid genome × 2 DNA strands). (**D**) Graphical depiction of the number of cumulative 6–4PPs and atypical photoproducts that become mutations in No PL yeast. Calculations were performed as described in panel (C) but with the median counts observed in *rad16∆ PHR1* (i.e. CPD Photolyase [CPD PL]) yeast, which should reflect all mutations other than CPDs, and the total number of lesions attributed to 6–4PPs and atypical PPs deduced from alkaline gel electrophoresis.

While our data so far indicate that not only CPDs but also 6–4PPs and atypical photoproducts contribute to UV mutagenesis, a key unanswered question is how frequently each class of photoproducts gives rise to a mutation. To estimate the mutagenic potential of each UV photoproduct, we measured the number of UV photoproducts that are induced by the total dose of UV light used for the WGS experiments. As each yeast isolate was exposed to 15 doses of ∼150 J/m^2^ of predominantly UVB light, giving a total dose of 2250 J/m^2^, we exposed the diploid *rad16*∆ No PL cells to 2250 J/m^2^, and measured the number of CPDs by treating the isolated genomic DNA with T4 endonuclease V (T4 endoV), which specifically cleaves CPDs, and analyzing the resulting digested DNA by alkaline gel electrophoresis (Fig. 7A). Alkaline gel analysis of the T4 endoV digested DNA indicated that this UV exposure induced ∼1.26 CPDs per kilobase (kb) of DNA (Fig. 7A and B). Since this technique measures the frequencies of CPDs per DNA strand, this indicates a total of ∼60 000 CPDs were induced over the 15 exposures (i.e. 1.26 CPDs/kilobases × 12 070 kilobasepairs/haploid genome × 2 haploid genomes/diploid × 2 bases/basepair = ∼60 000 CPDs). Analysis of the difference in mutation counts in the CPD PL isolates relative to the No PL control (Fig. 1C) indicate that CPDs were responsible for ∼398.5 mutations. This suggests that only 0.66% of CPDs (i.e. 398.5 mutations due to CPDs / ∼60 000 CPDs) caused mutations (Fig. 7C), even in GG-NER deficient *rad16*∆ yeast cells.

To estimate the mutation potential of non-CPD photoproducts (i.e. 6–4PPs and atypical photoproducts), we digested isolated genomic DNA from *rad16*∆ No PL diploid cells with ultraviolet damage endonuclease (UVDE) and analyzed the resulting products by alkaline gel electrophoresis (Fig. 7A). We and others have previously shown that UVDE cleaves a broad spectrum of UV photoproducts, including CPDs, 6–4PPs, and atypical photoproducts. To specifically measure the number of non-CPD photoproducts, we first incubated the isolated genomic DNA with recombinant CPD photolyase in the presence of UVA light to specifically repair CPD lesions prior to UVDE digestion. The results indicated that 2250 J/m^2^ of predominantly UVB light induces an average of 0.26 non-CPD photoproducts per kilobase of DNA (Fig. 7C). A similar calculation (see above) indicates that a total of ∼12 500 non-CPD photoproducts (i.e. 6–4PP and atypical photoproducts) are induced by the 15 cumulative UVB exposures. Analysis of the mutation counts in the CPD PL isolates (Fig. 1C), which contain mutations induced by non-CPD photoproducts, indicate that 6–4PPs and atypical photoproducts caused 274.5 mutations. This suggests that 2.18% (i.e. 274.5/12 500 = 2.18%) of the 6–4PPs and atypical photoproducts induced during the 15 UV exposures caused mutations (Fig. 7D).

## Discussion

**Figure 8.**
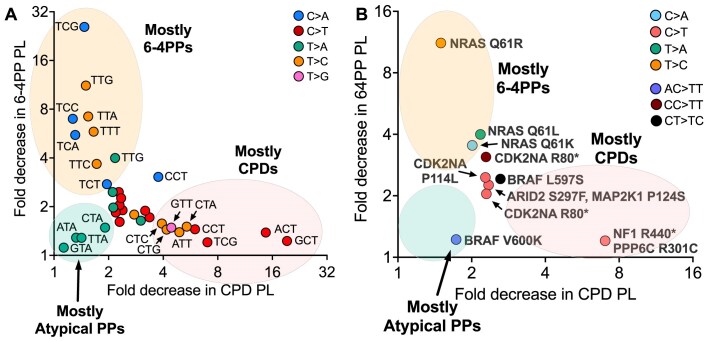
Role of CPDs and 6–4PPs in causing different UV mutation classes. (**A**) Graph depicting the fold decrease in mutations per isolate for each substitution class and trinucleotide context in the GG-NER deficient (i.e. *rad16*∆) CPD photolyase (CPD PL, *x*-axis) and 6–4PP photolyase (6–4PP PL, *y*-axis) strains relative to the No photolyase (No PL) control. Only trinucleotide contexts with a minimum of 70 mutations in the No PL data set are shown and each mutation class is labeled with a unique color (e.g. C > A mutations are blue, C > T mutations are red, etc.). Mutations lying close to the *x*-axis (indicated by light red shading) are likely derived predominantly from CPDs, while mutations lying close to the *y*-axis (indicated by light orange shading) are likely derived predominately from 6–4PPs. In contrast, mutations with low fold decreases in both CPDs and 6–4PPs (e.g. T > A mutations in NTA contexts, indicated by light green shading) likely represent mutations derived from atypical photoproducts. (**B**) Same as panel (A) except for mutations classes associated with a subset of recurrent driver mutations in melanoma that were also enriched in passaged No PL yeast. Mutation classes are designated by color (e.g. C > A mutations are light blue, AC > TT mutations are light purple, etc.) and the mutant allele of the driver mutation is indicated next to each. Trinucleotide contexts associated with C > T mutations are TCC (ARID2 S297F, MAP2K1 P124S), TCG (NF1 R440*, PPP6C R301C), CCC (CDKN2A P114L), CCG (CDKN2A R80*). The trinucleotide context for the NRAS Q61L and Q61R T > A and T > C mutations (respectively) is TTG, and the trinucleotide context for the NRAS Q61K mutation is ACA. Extended contexts for the double bases substitutions are not shown. Recurrent melanoma driver mutations were obtained from [12, 63].

UV light causes mutations by inducing multiple types of DNA damage. While UV-induced CPDs are the most common form of DNA damage, and are presumed to be the primary cause of UV signature C > T and CC > TT substitutions, UV exposure also induces less abundant 6–4PPs and rare atypical purine-containing photoproducts. We previously used WGS to discover that UV exposure in yeast cells induces not only the C > T and CC > TT mutations observed in human skin cancers, but also non-canonical C > A, T > C, T > A, and AC > TT substitutions [18–21], which are linked to a number of recurrent driver mutations in melanoma (e.g. *BRAF* V600E and V600K, *NRAS* Q61R, Q61K, Q61L, etc.). However, the UV photoproducts responsible for causing these non-canonical mutation classes were previously unclear. Here, we have used WGS of yeast strains expressing different combinations of CPD and/or 6–4PP photolyases (PL) to define the genomic mutation spectra of CPDs, 6–4PPs, and atypical photoproducts. Our results indicate that both CPDs and 6–4PPs make distinct but overlapping contributions to the overall UV mutation spectrum, and that 6–4PPs and atypical photoproducts are likely responsible for many non-canonical mutation classes associated with driver mutations in melanoma (Fig. 8).

Of the two lesion-specific photolyases, the photoreactivation with CPD PL had the greater overall effect on mutagenesis, reducing the number of accumulated mutations to ∼40% of that accumulated in yeast with no photolyases. This suggests that CPDs are responsible for the majority of UV-induced mutations, in agreement with previous findings [25–28]. Canonical C > T substitutions in dipyrimidine sequences constituted the greatest portion of CPD-induced mutations and were most abundant in TCN and NCT contexts. However, there were still considerable numbers of C > T substitutions in dipyrimidine sequence contexts in the CPD PL yeast isolates, particularly in CC and TC sequences. Our data suggest that these residual C > T substitutions may be caused by 6–4PPs, since photoreactivation with 6–4PP PL alone also decreases the frequency of C > T substitutions, and photoreactivation in cells expressing both photolyases eliminates essentially all C > T substitutions. In addition to C > T substitutions, CPDs also cause non-canonical mutation classes in yeast, including T > C substitutions, particularly in CT dipyrimidines, and T > A and T > G substitutions in TT dipyrimidines. Although CPDs cause the majority of UV-induced mutations in yeast, our data suggest that fewer than 1% of CPDs induced by the cumulative dose of UV light used in the WGS experiments result in mutations (Fig. 7). This finding is consistent with previous studies indicating that TT dimers, which are the most abundant form of CPDs, tend to be bypassed in an error-free manner by translesion (TLS) DNA polymerases [42–44]. Our analysis suggests that cytosine-containing CPDs are also frequently replicated in yeast without causing mutations, potentially either by TLS polymerases like Rad30/DNA Polymerase eta [45] or via a template-switch mechanism [46].

Most of the remaining ∼40% of UV-induced mutations in yeast that occur independent of CPDs are instead caused by 6–4PPs. Our data indicate that 6–4PPs are especially prone to cause T > C substitutions at the 3′ position in TT dipyrimidines. This is consistent with previous studies suggesting that 6–4PPs in TT sequence contexts in yeast are especially mutagenic due to misinsertion of a guanine nucleotide opposite the 3′T by yeast DNA polymerase eta (i.e. Rad30), resulting in a T > C mutations [47]. Moreover, our data indicate that less common C > A substitutions in dipyrimidine sequence contexts, especially TC dinucleotides, are also caused by 6–4PPs in UV-irradiated yeast. UVA light has also been reported to indirectly cause C > A/G > T substitutions through the induction of 8-oxoguanine and other oxidative lesions [48–50]. While this mechanism is likely responsible for causing most C > A substitutions in sunlight-exposed human skin cancers, the relatively low UVA dose used for photoreactivation (roughly ∼50 kJ/m^2^ of UVA light) is unlikely to cause such mutations in these experiments. For example, we have previously shown that 15 exposures to a much higher dose of UVA alone (i.e. 200 kJ/m^2^ of UVA light) induced only ∼6 mutations per isolate in yeast cells and had little effect on yeast survival [19]. Moreover, there were very few C > A substitutions in the 6–4PP PL and Both PL isolates, despite these cells being exposed to UVA photoreactivation. Exposure to UVA light can also convert 6–4PPs to a related lesion known as Dewar valence isomers [51], which could also contribute to the spectrum of UV-induced mutations reported herein. Given that 6–4PPs occur at < 20% the frequency of CPDs (Fig. 7A and B), our mutation data imply that 6–4PPs are ∼3–4 times more mutagenic than CPDs (compare Fig. 7C and D).

While our data indicate that 6–4PPs cause many UV mutations in yeast, the role of 6–4PPs in UV mutagenesis in mammalian cells is controversial. A number of previous studies using photolyases in conjunction with mutational reporter genes concluded that 6–4PPs do not significantly contribute to UV mutagenesis in mammalian cells [25–27]. This discrepancy with our findings could in part be due to species-specific differences in the fidelity DNA polymerase eta [18] and potentially the role of other mammalian translesion DNA polymerases (e.g. DNA polymerase iota, etc.) in bypassing 6–4PPs. Alternatively, 6–4PPs may be more mutagenic in GG-NER deficient (i.e. *rad16*∆) cells, since they are repaired more rapidly than CPDs [52], particularly in mammalian cells (e.g. [53]). Consistent with this hypothesis, whole exome sequencing of UVB-irradiated *XPC^−/−^* skin fibroblasts, which are deficient in GG-NER, revealed not only UV-induced C > T substitutions but also T > A, T > C, and C > A mutations [54]. Moreover, a previous study that transfected a UV-irradiated and photoreactivated plasmid into NER-deficient human cells found that 6–4PPs significantly contribute to both UV-induced C > T and T > C substitutions, consistent with our results [28]. We have previously shown that NER-proficient WT cells have a very similar UV mutation spectrum to *rad16*∆ cells, with abundant T > C substitutions in TTN sequence contexts and C > A substitutions in TCN contexts [19, 20]. Since our data indicate that these mutation classes are primarily caused by 6–4PPs (see below), these results suggest that 6–4PPs play a similarly important role in UV mutagenesis in NER-proficient yeast cells.

Both CPDs and 6–4PPs contribute to canonical C > T substitutions and non-canonical C > A, T > A, and T > C substitutions. However, comparison of the fold decrease in UV mutations in the photoreactivated CPD PL or 6–4PP PL strains (each relative to the No PL control) reveals that there are certain mutation classes and sequence contexts that are almost exclusively caused by just CPDs or 6–4PPs (Fig. 8A). For example, C > T in NCT sequence contexts (e.g. ACT, GCT, CCT) and T > C substitutions in CTN sequence contexts (e.g. CTA, CTC, and CTG) are significantly depleted by CPD photolyase, but minimally affected by the 6–4PP photolyase (Fig. 8A). These data indicate that mutations occurring in CT dipyrimidines are primarily derived from CPDs. This observation is consistent with previous studies indicating that 6–4PPs form very infrequently at CT dinucleotides, while CPDs occur much more abundantly in this sequence context [5, 8, 10]. On the other hand, C > A mutations in TCN sequence contexts (e.g. TCA, TCC, and TCG) and T > C substitutions in TTN sequence contexts (i.e. TTA, TTC, TTG, and TTT) are almost exclusively caused by 6–4PPs (Fig. 8A). Notably, some sequence contexts show different contributions from CPDs or 6–4PPs depending on the mutation type. For example, UV mutations in TCG sequence contexts are primarily caused by 6–4PPs if they are C > A substitutions, but by CPDs if they are C > T substitutions (Fig. 8A). Elevated CPD-induced C > T substitutions in this sequence context may be a consequence of especially rapid CPD deamination in TCG sequences [55].

There is a distinct cluster of mutation classes, comprised of T > A substitutions in NTA sequence contexts (i.e. ATA, CTA, GTA, and TTA), that are impacted least by CPD PL and 6–4PP PL photoreactivation (Fig. 8A). These mutation classes are unusual in that a subset occur in non-dipyrimidine sequence contexts (i.e. ATA and GTA) and each showed reverse transcriptional asymmetry, with elevated T > A substitutions on the TS of yeast genes instead of the NTS. These and other lines of evidence suggest that these are in actuality A > T substitutions caused by atypical thymine-adenine TA photoproducts (i.e. TAN sequence context on opposite strand). Consistent with this hypothesis, substantial numbers of A > T substitutions in these sequence contexts are still present in yeast photoreactivated with both photolyases, unlike essentially all other classes of UV-induced single base substitutions. Moreover, our prior analysis of UV-induced mutations in *rad14*∆ yeast cells (which are deficient in both NER pathways) revealed enrichment of T > A substitutions after photoreactivation with both photolyases [33, 56]. However, this prior study also found that in the photoreactivated *rad14*∆ cells, there were abundant C > T substitutions that were highly enriched on the TS of yeast genes, likely due to CPDs and/or 6–4PPs that were protected from photolyase repair by persistent RNA polymerase II stalling at the lesion in the absence of TC-NER [33, 56]. The analysis reported here supports this hypothesis, as we did not observe significant numbers of C > T substitutions in *rad16*∆ cells photoreactivated with both photolyases, presumably due to ongoing TC-NER in these strains. A previous study in NER-deficient mammalian cells found an enrichment of T > A mutations in NTA contexts after transfection of UV-irradiated plasmids that had been similarly photoreactivated with both CPD and 6–4PP photolyases [28], consistent with our results. While UV-induced thymine-adenine (TA) photoproducts were discovered >40 years ago [24], and genome-wide mapping data indicate that TA photoproducts are more abundant than CC and CT 6–4PPs [10, 20], the extent to which TA photoproducts contribute to UV mutagenesis is still being actively investigated.

Our results indicate that TA photoproducts cause many UV-induced T > A substitutions in yeast. One caveat to our model is that T > A substitutions in NTA sequence contexts are significantly decreased, albeit marginally (<2-fold), in the CPD PL and 6–4PP PL isolates, as well as in yeast cells expressing both photolyases. One possible explanation is that a subset of the T > A substitutions in these sequence contexts are caused by CPDs and/or 6–4PPs. In cases where the mutation occurred in a non-dipyrimidine sequence context (i.e. ATA or GTA), this could be a result of “collateral damage” caused by error-prone replication by a translesion DNA polymerase bypassing a neighboring CPD or 6–4PP [57]. However, an alternative possibility is that rapid removal of one or both of the major classes of UV damage (i.e. CPDs and 6–4PPs) by photolyase(s) could indirectly promote repair of other UV photoproducts (i.e. TA photoproducts) or mutation avoidance of these lesions by post-replication repair (PRR) pathways such as via a template switch mechanism [46] or error-free bypass by TLS polymerases (e.g. Rad30) [42, 45]. In photoreactivated strains, NER and PRR pathways have fewer lesions to repair or bypass, so these pathways should in principle be more efficient at repairing or bypassing (in an error-free manner) atypical photoproducts, potentially explaining the observed decrease in T > A mutations. We have tried to mitigate this effect in our photoreactivation experiments by utilizing GG-NER deficient cells, lacking the Rad16 subunit of the Rad16/Rad7/Elc1 complex, which is the functional counterpart in yeast of the human UV-DDB complex. However, any residual GG-NER activity in the *rad16*∆ strains [58], along with ongoing TC-NER and PRR, may be responsible for the observed decrease in T > A substitutions in NTA contexts in photoreactivated yeast cells. It is also possible that selection against deleterious mutations (e.g. in essential genes) could in principle affect the genome-wide mutation spectra. However, preliminary experiments examining sporulation of the Both PL diploid cells after UV passaging indicated that some of the Both PL isolates contained one or more recessive lethal mutations, resulting in inviable haploid spores (Supplementary Fig. S6). That these lethal mutations were maintained during the UV passaging experiment, presumably because the diploid cells retained one functional copy of the essential gene, suggests that selection, at least for recessive, loss-of-function mutations, did not significantly impact the mutation spectra reported herein.

Whereas CPDs are the primary cause of C > T single base substitutions, we find that they cause roughly half of CC > TT double base substitutions (DBS) and the rest originate from 6–4PPs (Fig. 8B). The role of 6–4PPs in these DBS can potentially explain why CC > TT mutations are highly elevated in tumors of XP-C patients, who are deficient in GG-NER, but not in the tumors of XP-E patients [49], who have a defect in repair of CPDs but not 6–4PPs. UV exposure also induces a wide variety of non-canonical double base substitutions in yeast. Many of these occur in dipyrimidine sequences (e.g. CT > TA, CT > TC, etc.) and our analysis of the spectrum of DBS in photolyase-expressing cells indicate they are caused by CPDs, 6–4PPs or both. However, we also observed other DBS occurring in purine-pyrimidine contexts, most notable AC > TT mutations. Like T > A single base substitutions, AC > TT mutations were the predominant DBS in yeast photoreactivated with both CPD and 6–4PP photolyases (i.e. 31 of 56 total DBS in the Both PL isolates, Fig. 6D). We have previously shown that AC > TT substitutions are the most frequent class of DBS in yeast exposed to UVC light, and found that they are induced by the mutagenic bypass of an unknown photoproduct by DNA polymerase eta [20, 21]. Since AC > TT mutations are enriched on the NTS of yeast genes, and are the most resistant type of DBS to photoreactivation by CPD and 6–4PP photolyases, we hypothesize that they arise from a previously uncharacterized atypical adenine-cytosine (AC) photoproduct.

Many of the non-canonical single and double base substitutions characterized in this study cause driver mutations in skin cancer such as melanoma. For example, AC > TT substitutions cause the oncogenic *BRAF* V600K mutation, which occurs in ∼5%–10% of cutaneous melanomas [12, 59, 60]. Epidemiological studies indicate that *BRAF* V600K is significantly associated with melanomas occurring in sun-exposed anatomical locations (i.e. head and neck) and geographical locations with high ambient sunlight (e.g. Florida, Australia), consistent with a UV origin [61, 62]. However, the mechanism by which UV light induces a non-dipyrimidine DBS was previously unclear. Our photoreactivation data in yeast suggest that AC > TT mutations are caused by an atypical purine-containing photoproduct (Fig. 8B). A similar mechanism is likely responsible for the less common *BRAF* V600R mutation, which is caused by a related AC > CT substitution, also observed in UV-exposed yeast cells. The most common oncogenic *BRAF* mutation (i.e. V600E) is caused by a T > A substitution in a GTG sequence context; however, there were too few of this mutation class in UV-exposed yeast cells to characterize its origin. Other recurrent driver mutations in melanoma include the *NRAS* Q61L and Q61R mutations, which are caused by T > A and T > C substitutions, respectively, in a TTG sequence context. Our data indicate that both of these mutation classes are primarily caused by 6–4PPs in yeast (Fig. 8B). Taken together, these findings suggest that 6–4PPs and atypical photoproducts may play important roles in causing key driver mutations in melanoma.

In conclusion, WGS of UV-irradiated yeast cells photoreactivated with different combinations of photolyase enzymes indicate that 6–4PPs and atypical photoproducts have significant roles in UV mutagenesis. Many of the non-canonical mutation classes induced by UV light, including C > A, T > A, T > C, and AC > TT substitutions, are primarily induced by either 6–4PPs or atypical UV photoproducts, such as TA photoproducts. Importantly, our data suggest that a number of the recurrent driver mutations in the *NRAS* and *BRAF* oncogenes in cutaneous melanoma are primarily induced by 6–4PPs or atypical photoproducts in UV exposed yeast. These findings have potentially important implications for our understanding of the molecular etiology of key oncogenic mutations in melanoma.

## Funding

This research was supported by National Institute of Environmental Health Sciences grants R01ES028698, R01ES032814, R21ES035139, and R21ES035888. Funding to pay the Open Access publication charges for this article was provided by the NIH.

## Supplementary Material

gkag482_Supplemental_Files

## Data Availability

The sequencing data used to identify UV-induced mutations in whole genome sequenced yeast are available at NCBI Sequence Read Archive (SRA; https://www.ncbi.nlm.nih.gov/sra) under BioProject accession number PRJNA1371130. Custom software code used to analyze mutation data can be found in Zenodo at https://doi.org/10.5281/zenodo.17957176. A list of identified mutations can be found in Supplementary Data S2.
