# Supplementary material for: Genome-wide mutation spectra of canonical and atypical UV photoproducts in S. cerevisiae
Source: Nucleic Acids Res. 2026 May 20;54(10):gkag482. doi: 10.1093/nar/gkag482 (PMC13187834; doi:10.1093/nar/gkag482)
Supplement: gkag482_Supplemental_Files [file gkag482_supplemental_files.zip › Laughery_etal_SupplementaryData_revision.pdf]

Supplementary Materials

Supplementary Methods

Tetrad Dissection of UV Passaged Yeast Isolates

UV exposed/passaged isolates of the Both PL (both photolyase) yeast strain that had been saved as frozen permanent stocks were patched to YPD medium and allowed to incubate at 30°C to allow growth on the plate. Each isolate was then patched to sporulation medium (final concentration 0.05% potassium acetate, 0.05% yeast extract, 0.025% dextrose, 2% agar) and incubated at room temperature for approximately one week.

Cells from sporulation plates were resuspended in water, adding 0.2 volumes of Zymolyase-100T (Amsbio), incubated for five minutes at 30°C, and 1.7 volumes of water was added. Manual tetrad dissection was performed using a Nikon Labophot-2 dissection microscope. Four separate tetrads were dissected from each passaged isolate on YPD plates in a 4 x 4 array and allowed to grow several days at 30°C to allow spores to grow. For isolates that uniformly produced <4 viable spores (i.e. none of the four dissected tetrads yielded 4 colonies on the plate), re-inspection by microscopy was performed on selected dissections to confirm the original placement of spores.

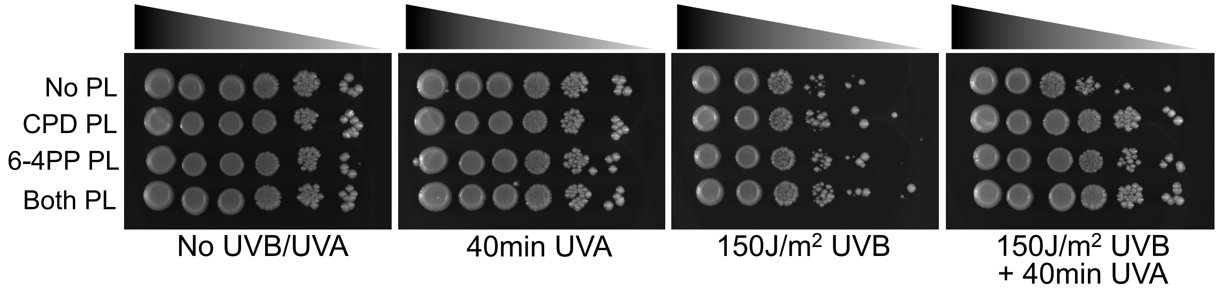

Supplementary Figure S1. Overnight cultures of each diploid passaging strain were spotted in 10-fold serial dilutions on YPD media plates (arrows above each panel indicate plated cell density decreasing from left to right) and allowed to dry before exposing them to 150 J/m^2 of UVB followed by 40 minutes of photoreactivation with UVA (far right panel). Additional controls were performed with no UV exposure, UVA exposure alone, and UVB exposure alone (shown left to right, respectively). UVA irradiation was performed with lids on the petri dish to block any trace UVB that might be emitted by the UVA lamps. No photolyase strain (No PL) is *rad16Δ phr1Δ*; CPD photolyase (CPD PL) yeast strain is *rad16Δ PHR1*; 6-4PP photolyase (6-4PP PL) yeast strain is *rad16Δ phr1Δ dPhr(6-4)* (i.e., *Drosophila* 6-4PP photolyase); and both photolyases (Both PL) yeast strain is *rad16Δ PHR1 dPhr(6-4)*.

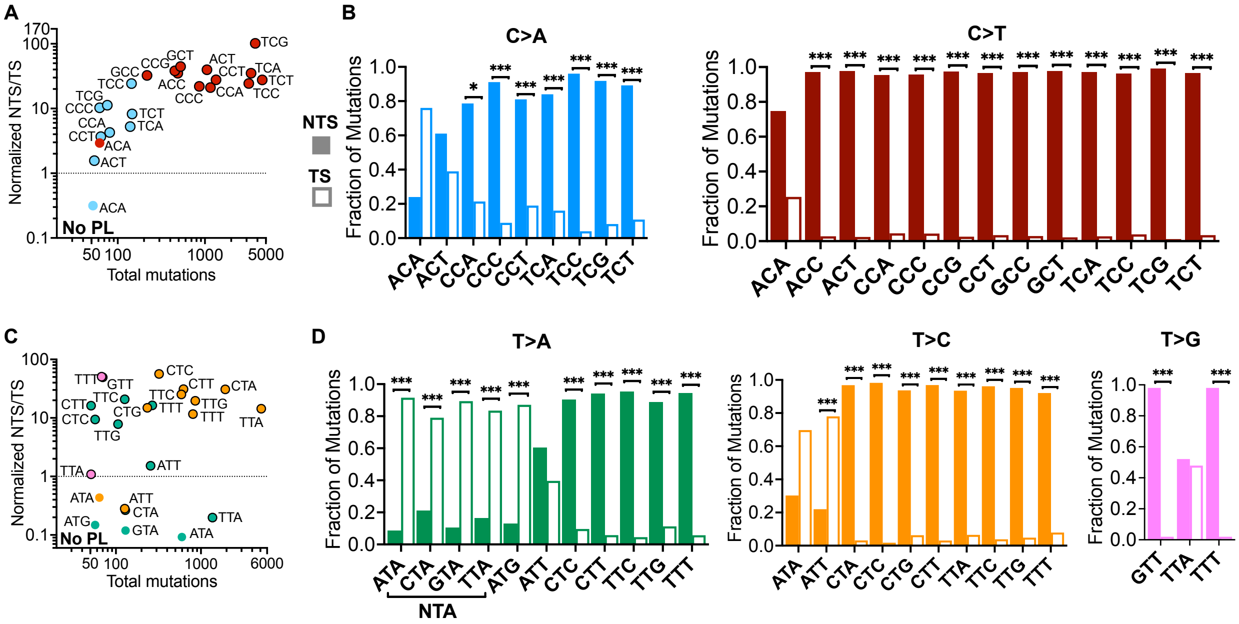

Supplementary Figure S2. Mutations in No Photolyase (No PL) passaged yeast (i.e., *rad16*Δ *phr1*Δ) show very strong transcriptional strand asymmetry. **A.** C>A (blue) and C>T (red) mutations are enriched on the nontranscribed strand (NTS) relative to the transcribed strand (TS) in No PL passaged yeast. Trinucleotide contexts with a minimum of 50 total mutations of a single mutation class are shown and colored by that substitution class. Contexts containing dipyrimidines are indicated with a black outline. **B.** Bar graphs of the fraction of mutations observed on the NTS (solid bars) and TS (outlined bars) for C>A (blue) and C>T (red) substitutions shown in panel A. **P<0.001 based on chi squared test with Bonferroni correction. **C.** NTS/TS asymmetry for T>A (green), T>C (orange), and T>G (pink) trinucleotide contexts plotted as described in (A) **D.** Bar graphs of T>A, T>C, and T>G mutations, depicted as described in **B**. While most mutations show typical transcription strand asymmetry, T>A mutations in NTA contexts and certain T>C mutations in the ATT context show reverse strand asymmetry, indicating the that UV-induced lesion is occurring in the complementary trinucleotide context on the NTS. ***P<0.001 based on chi-square test with Bonferroni correction.

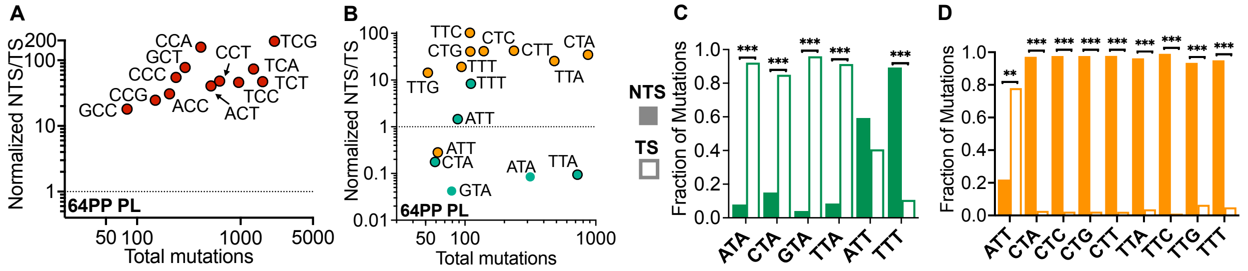

Supplementary Figure S3. A. C>T (red) mutations are enriched on the non-transcribed strand (NTS) relative to the transcribed strand (TS) in 6-4PP photolyase (6-4PP PL, *rad16Δ phr1Δ dPhr(6-4)*) passaged yeast and are the most abundant class of mutations originating from cytosine bases observed in this strain. Trinucleotide contexts with a minimum of 50 total mutations of a single mutation class are shown and colored by that substitution class. Contexts containing dipyrimidines are indicated with a black outline. **B.** NTS/TS asymmetry for T>A (green) and T>C (orange) trinucleotide contexts plotted as described in (**A**)**. C.** Bar graphs of the faction of mutations occurring on the NTS (shaded) vs TS (outlined) for T>A (green) and **D.** T>C (orange) trinucleotide contexts with at least 50 cumulative mutations. Most T>C mutations show traditional transcriptional strand asymmetry, with significant enrichment of mutations occurring on the NTS. T>A mutations in NTA contexts show reverse strand asymmetry. **P<0.01, ***P<0.001 based on chi-square test with Bonferroni correction.

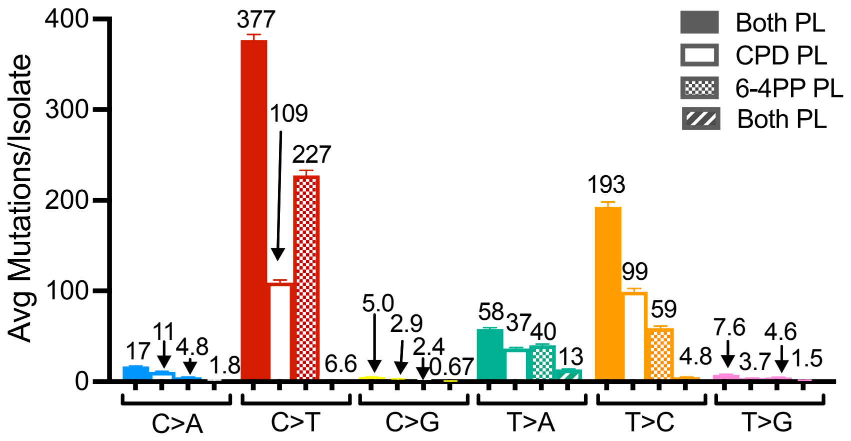

Supplementary Figure S4. Summary of the single base substitutions occurring in each passaged yeast strain. Mutations are colored by substitution class and average mutations/class are indicated, with bars representing standard error of the mean (SEM). Note that T>A mutations constitute the most abundant mutation class in yeast expressing both CPD and 6-4PP photolyases (Both PL). No photolyase strain (No PL) is *rad16Δ phr1Δ*; CPD photolyase (CPD PL) yeast strain is *rad16Δ PHR1*; 6-4PP photolyase (6-4PP PL) yeast strain is *rad16Δ phr1Δ dPhr(6-4)* (i.e., *Drosophila* 6-4PP photolyase); and both photolyases (Both PL) yeast strain is *rad16Δ PHR1 dPhr(6-4)*.

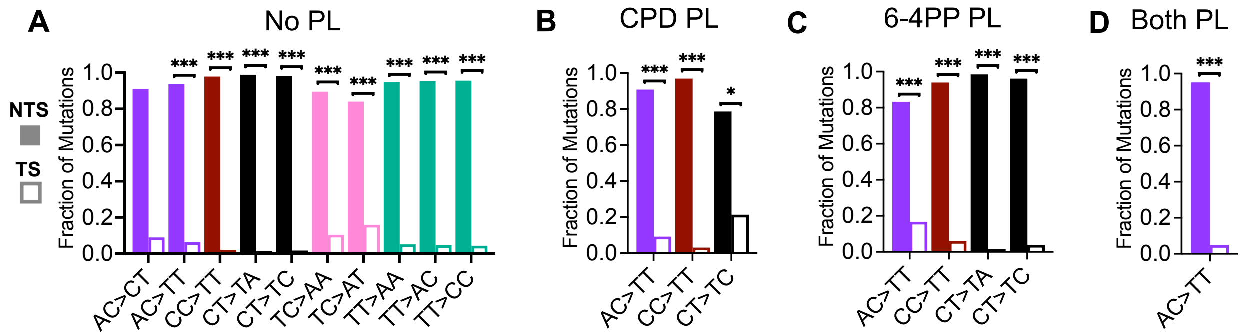

Supplementary Figure S5. Tandem mutations resulting from UVB/UVA passaging are significantly enriched on the non-transcribed strand (NTS) of yeast genes. Bar graphs of the fraction of mutations observed on the NTS (solid bars) and TS (outlined bars) for tandem mutation classes with a minimum of 20 cumulative mutations in **A.** No PL, **B.** CPD PL, **C.** 6-4PP PL, and **D.** Both PL yeast. No photolyase strain (No PL) is *rad16*Δ *phr1*Δ; CPD photolyase (CPD PL) yeast strain is *rad16*Δ *PHR1*; 6-4PP photolyase (6-4PP PL) yeast strain is *rad16*Δ *phr1*Δ *dPhr(6-4)* [i.e., *Drosophila* 6-4PP photolyase gene)]; and both photolyases (Both PL) yeast strain is *rad16*Δ *PHR1 dPhr(6-4)*. Bars are colored according to the dinucleotide context from which they originate as shown in the mutation spectra of **Figure 6**. *P < 0.05, ***P < 0.001 based on a chi-square test with the Bonferroni correction.

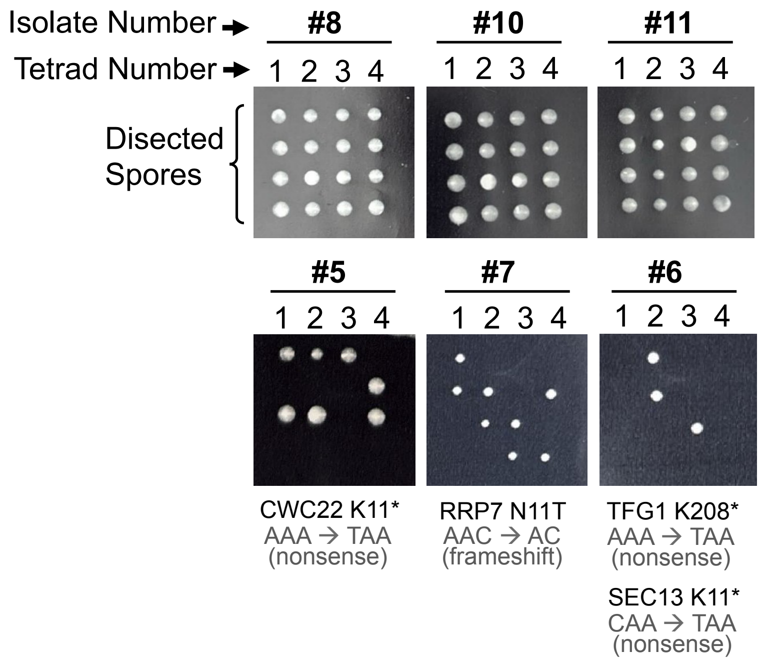

Supplementary Figure S6. Tetrad dissection of both photolyase (Both PL) passaged isolates (i.e., *rad16Δ PHR1 dPhr(6-4)*). Twelve isolates from Both PL yeast that had been exposed to 15 rounds of UVB exposure + UVA photoreactivation were sporulated, followed by dissection of four tetrads from each isolate to investigate the viability of haploid daughter cells. While each of the four haploid daughter cells were viable in many of these isolates (three examples shown in top panels), three of the twelve dissections resulted in ≤50% viability of daughter cells (bottom panels), indicating complementation of lethal mutations in the diploid strains. Analysis of coding mutations occurring in these isolates revealed that each had a minimum of one mutation resulting in a premature stop codon or frameshift mutation in an essential gene, as indicated below each panel, along with the nucleotide change that caused the codon change.

Supplementary Table S1: Yeast strains, plasmids, and oligonucleotides used in this study.

Strain	Description	Genotype
MP072	Haploid CPD PL strain	*MATa his3Δ1 leu2Δ0 met15Δ0 ura3Δ0 trp1::HIS3 rad16::TRP1* (previously described in Laughery, et al. 2020)
YML155	Diploid CPD PL strain (for passaging)	*MATa/α his3Δ1/his3Δ1 leu2Δ0/leu2Δ0 LYS2/lys2Δ0 met15Δ0/MET15 ura3Δ0/ura3Δ0 rad16::TRP1/rad16::LEU2* (previously described in Laughery, et al. 2020)
YML478	Diploid CPD PL strain (for strain construction)	*MATa/alpha his3-1/his3-1 leu2-0/leu2-0 LYS2/lys2-0 met15-0/MET15 ura3-0/ura3-0 TRP1/trp1::HIS3 rad16::TRP1/rad16::LEU2 leu2-ML84/leu2-ML84*
YML481	Diploid No PL strain with telomeric deletion	*MATa/alpha his3-1/his3-1 leu2-0/leu2-0 LYS2/lys2-0 met15-0/MET15 ura3-0/ura3-0 TRP1/trp1::HIS3 rad16::TRP1/rad16::LEU2 leu2-ML84/leu2-ML84 Chromosome XV 34kb truncation*
YML482	Diploid No PL strain with ORF-specific *phr1* deletion	*MATa/alpha his3-1/his3-1 leu2-0/leu2-0 LYS2/lys2-0 met15-0/MET15 ura3-0/ura3-0 TRP1/trp1::HIS3 rad16::TRP1/rad16::LEU2 leu2-ML84/leu2-ML84 phr1-ML84/phr1-ML84*
YML486	Diploid 64PP PL strain	*MATa/alpha his3-1/his3-1 leu2-0/leu2-0 LYS2/lys2-0 met15-0/MET15 ura3-0/ura3-0 TRP1/trp1::HIS3 rad16::TRP1/rad16::LEU2 leu2-ML84/leu2-ML84 phr1-ML84/phr1-ML84 + integrated pML157 [pTEF1-dPhr(6-4)]*
YML487	Diploid Both PL strain	*MATa/alpha his3-1/his3-1 leu2-0/leu2-0 LYS2/lys2-0 met15-0/MET15 ura3-0/ura3-0 TRP1/trp1::HIS3 rad16::TRP1/rad16::LEU2 leu2-ML84/leu2-ML84 + integrated pML157 [pTEF1-dPhr(6-4)]*
YML496	Haploid No PL strain	*MATa his3-1 leu2-0 met15-0 ura3-0 trp1::HIS3 rad16::TRP1 phr1-ML84*
YML497	Haploid Both PL strain	*MATa his3Δ1 leu2Δ0 met15Δ0 ura3Δ0 trp1::HIS3 rad16::TRP1 + integrated pML157 [pTEF1-dPhr(6-4)]*
YML498	Haploid 64PP PL strain	*MATa his3Δ1 leu2Δ0 met15Δ0 ura3Δ0 trp1::HIS3 rad16::TRP1 phr1-ML84 + integrated pML157 [pTEF1-dPhr(6-4)]*
**Plasmid**	**Description**	**Construction**
pML157	Integrating plasmid containing *Drosophila melanogaster phr(6-4)* [dPhr(6-4)] downstream of	Ligation of NheI/XhoI excised *D. melanogaster* 64PP photolyase gene from p64PLA5 (Addgene #67284) with SpeI/XhoI digested p405TEF1 (Addgene #15968)

	TEF1 promoter	
pML159	CRISPR/Cas9 plasmid targeting *PHR1*	Ligation of pML104 with OML327/OML328 guide oligonucleotides (Laughery, et al. 2015)
pML160	CRISPR/Cas9 plasmid targeting *LEU2*	Ligation of pML104 with OML341/OML342 guide oligonucleotides (Laughery et al. 2015)
**Oligo**	**Description**	**Sequence**
OML248	phr1 internal forward primer	GTGGAACCTCTGGGCTAAGTGTA
OML249	phr1 internal reverse primer	GCATTCCCATTGAGGTATATGGC
OML327	pML159 sgRNA oligo 1	GATCCGAAAAACGAGGAAGAAAAGGTTTTAGAGCTAG
OML328	pML159 sgRNA oligo 2	CTAGCTCTAAAACCTTTTCTTCCTCGTTTTTCG
OML330	dPhr(6-4) reverse primer	CACACCCTCTGCCTTGGCC
OML336	*TEF1* promoter forward primer	GCATCGCCGTACCACTTCAA
OML340	Repair template for CRISPR/Cas9 mediated *PHR1* deletion	GAAGTTATTGATATGAAGATGACGCTTCATAATGCTAG CTACAACCCAAAACCTTTAGTTGATTTAAAGCATAGTA GGGAGCGTGCTTTG
OML341	pML160 sgRNA oligo 1	GATCAATCACAGCCGAAGCCATTAGTTTTAGAGCTAG
OML342	pML160 sgRNA oligo 2	CTAGCTCTAAAACTAATGGCTTCGGCTGTGATT
OML343	Repair template for CRISPR/Cas9 mediated *LEU2* inactivation	CGTTGGTCAAGAAATCACAGCCGAAGCCATTAATTCTT AAAGCTATTTCTGATGTTCGTTCCAATGTCAAGTTCGAT TTCGAAAATCA
OML381	*PHR1* flanking forward primer	CTTGACGTGTAACACCCAGAGG
OML382	*PHR1* flanking reverse primer	GGTCTGGATTAAGATGGGCACA

Supplementary Table S2. Number of isolates from each yeast strain that were genome sequenced and analyzed further (i.e., passed quality control).

Yeast Strain	Replicate 1 isolates	Replicate 2 isolates	Total isolates
No PL	24	32	56
CPD PL	23	16	39
64PP PL	22	16	38
Both BL	24	15	39
